# Personalised management of community-acquired pneumonia and the role of Clinical Decision Support Software

**DOI:** 10.1136/bmjresp-2024-003096

**Published:** 2026-04-15

**Authors:** Christopher Hatton, Catherine Atkin, Suzy Gallier, Elizabeth Sapey

**Affiliations:** 1 Department of Inflammation and Ageing, School of Infection, Inflammation, and Immunology, College of Medicine and Health, University of Birmingham, Birmingham, UK; 2 NIHR Midlands Patient Safety Research Collaboration, University of Birmingham, Birmingham, England, UK; 3 PIONEER Health Data Research Hub in Acute Care, Department of Research Development and Innovation, University Hospitals Birmingham NHS Foundation Trust, Birmingham, UK

**Keywords:** Pneumonia, Respiratory Infection

## Abstract

Community-acquired pneumonia (CAP) is one of the most common causes of hospital admission and is associated with significant morbidity and mortality. National and international guidelines are available to guide the management of CAP, including antibiotic prescribing. However, these guidelines are often not adhered to and there is significant overprescribing of broad-spectrum antibiotics, contributing to the growing pandemic of antimicrobial resistance. Clinical Decision Support Software (CDSS) are electronic tools that use individual patient data to generate patient specific assessments or recommendations, that can then be acted on by the patient or clinical decision maker. This article reviews the evidence surrounding the initial management of CAP in hospital and considers the potential of CDSS to support CAP management.

## Introduction

 Community-acquired pneumonia (CAP) is one of the most common causes of admission to hospital in the UK, and accounts for over 100 000 admissions each year.^1^ It is associated with significant morbidity and mortality, with an in-hospital mortality rate of approximately 10%.^2^ In the UK, on average, over 25 000 people die each year secondary to pneumonia, and the death rate in the UK is the third highest in Europe.^3^ Admissions secondary to CAP are rising, readmission rates are high and the health economic consequences are significant.^4 5^ Therefore, it is unsurprising that the management of CAP has been highlighted in health policy and was the focus of a recent National Confidential Enquiry into Patient Outcome and Death.^1 6 7^ Despite its substantial impact, there has been significant underinvestment in CAP research relative to similarly burdensome diseases.^8^

Major contributors to CAP-associated morbidity and mortality include the direct consequences of infection, the host response to infection and harms secondary to antibiotic use. Harms secondary to the use of antibiotics include adverse side effects which affect around 20% of hospitalised patients treated with antibiotics,^9^ increased susceptibility to hospital-acquired infections^10^ and increasing antimicrobial resistance at the individual and population level. International guidelines are available to support the management of CAP.^11 12^ A fundamental aim of these guidelines is to standardise antibiotic prescribing and maximise the potential benefit of antibiotic use in CAP while minimising the potential harms. However, in clinical practice, adherence to guidelines is poor and there is significant overprescribing of broad-spectrum antibiotics, contributing to the growing pandemic of antimicrobial resistance.^2 13 14^ Addressing this growing threat is high on the agenda of the UK Department of Health and Social Care, who have recently published a 5-year action plan to support the UK government’s vision to control antimicrobial resistance by 2040.^15^ Within this action plan, there is a commitment to the use of Clinical Decision Support Software (CDSS) to support antibiotic prescribing decisions of frontline clinical decision makers.

**Figure 1:**
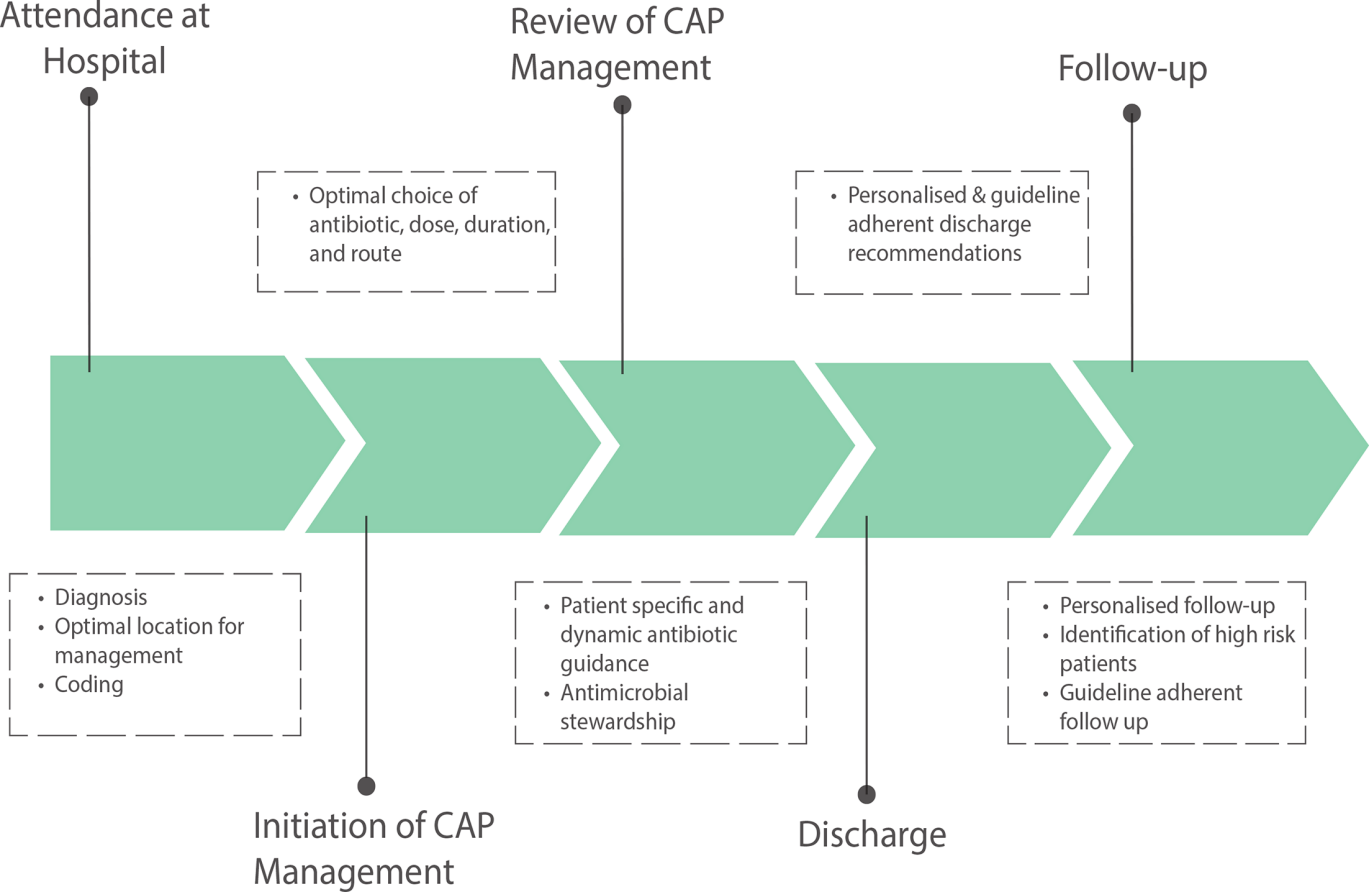
Flow diagram to illustrate the potential use of CDSS for patients admitted to hospital with CAP. CAP, community-acquired pneumonia.

CDSS are defined as software that is designed to be a direct aid to clinical decision making, in which the characteristics of an individual patient are matched to a computerised clinical knowledge base, and patient-specific assessments or recommendations are presented to the clinician or patient for a decision.^16^ Software meeting this definition is heterogeneous. They include comparatively simple systems that match patient information to a knowledge base, such as a clinical guideline, to provide an output for the clinical decision maker or patient. It also includes software that uses complex statistical algorithms, such as machine learning, to provide patient-specific outputs.^17^ CDSSs have the potential to influence the management of CAP across the entire patient journey from initial presentation to follow-up, as shown in figure 1. This article will discuss the evidence surrounding the initial management of CAP and explore how CDSS could be used.

## Community-acquired pneumonia

The diagnosis of CAP is generally accepted to require the presence of symptoms of a lower respiratory tract infection and radiological evidence of new pulmonary infiltrates in most international guidelines.^11 18^ Once the diagnosis has been confirmed, two major decisions are required:

1. The optimal location for patient care. This includes outpatient or hospital inpatient (within the intensive care unit (ICU) or outside the ICU).
2. The optimal empirical antibiotic(s) for individual patients, pending microbiological diagnosis.

### Where to care for patients with CAP?

Determining the optimal location to care for patients with CAP is multifaceted and dependent on several factors including the severity of CAP, individual patient medical and social circumstances, likelihood of deterioration or development of complications, and any advanced directives which limit invasive interventions. There are several benefits to outpatient management, including reduced costs, reduced exposure to hospital-acquired infections and for most patients, outpatient treatment is preferred.^19^ These benefits need to be carefully weighed against the safety and appropriateness of outpatient treatment.

Currently, most major international guidelines recommend basing this decision on pneumonia severity assessment scores, together with clinical judgement.^11 18 20^ Several CAP severity assessment scores exist and are used to provide an objective assessment of disease severity, most commonly using 30-day mortality risk as the indicator of severity. Scores recommended by major international CAP management guidelines include the Pneumonia Severity Index (PSI), recommended by the Infectious Diseases Society of America (IDSA)/American Thoracic Society (ATS) guidelines and CURB-65 (confusion, urea >7mmol/L, respiratory rate ≥30, blood pressure with systolic <90 mmHg or diastolic ≤60 mmHg, age ≥ 65)/CRB-65 (confusion, respiratory rate ≥30, blood pressure with systolic <90 mm Hg or diastolic ≤60 mmHg, age ≥ 65), recommended by the British Thoracic Society (BTS) and National Institute for Health and Care Excellence (NICE) guidelines in the UK and within European guidelines.^11 18 20 21^ A comparison of the components used in each of these scores is provided in table 1. The ATS/IDSA guidelines recommend the PSI on the basis that it identifies a higher proportion of low-risk patients and may have greater discriminatory power than CURB-65.^22^ There is also more substantial evidence to suggest that the PSI is safe and effective in selecting patients for outpatient care.^23^ Other major international guidelines recommend CURB-65 on the basis of lesser complexity and similar predictive performance.^24^ However, few studies compare the effectiveness or safety of CURB-65 and the PSI directly. A recent retrospective study in the Netherlands suggested that 30-day mortality may be lower in hospitals that use CURB-65 to assess disease severity, compared with those that use PSI.^25^ However, there are several confounders that are not adjusted for in this study, including pneumonia severity, and residual confounding may account for the observed results.

**Table 1:** Comparison of community-acquired pneumonia severity scoring system components

Component	Pneumonia Severity Index	CURB-65/CRB-65
Demographics	Age Sex Nursing home residency	Age
Physical examination/observations	Mental status Respiratory rate Systolic blood pressure Temperature Pulse rate	Confusion (new onset) Respiratory rate Systolic and diastolic blood pressure
Laboratory findings	Urea Blood pH Glucose Haematocrit Sodium Arterial PaO2	Urea*
Radiographic findings	Presence of pleural effusion	
Comorbidities	Neoplastic disease Liver disease Congestive heart failure Cerebrovascular disease Renal disease	

This table provides an overview of the components included in two of the major severity assessment tools for CAP: CURB-65, and the PSI. These scores both indicate the severity of CAP based on 30-day mortality.

* CRB-65 includes all parameters in CURB-65 except urea. It is used more frequently in primary care where laboratory investigations are less frequent.

CRB-65, A severity score for pneumonia that uses the following elements: confusion (new onset), respiratory rate, blood pressure, and age; CURB-65, A severity score for pneumonia that uses the following elements: confusion (new onset), urea, respiratory rate, blood pressure, and age; Pa02, Partial pressure of oxygen.

While severity assessment scores offer an objective estimation of disease severity, there are several limitations. First, their utility is reliant on the assumption that 30-day mortality is the most important factor to determine the location of care. In reality, this decision is nuanced and should be based on a holistic assessment of the potential of an individual to benefit from inpatient care, versus the potential of an individual to be harmed by inpatient care. Severity assessment scores provide a crude estimation of only one aspect of this decision making process. Second, there is an assumption that severity assessment scores are equitable and accurate for all populations, but this assumption does not hold true. Both scores have been shown to be less accurate in both younger and older patients.^26^ Finally, their benefit depends on utilisation by frontline clinical decision makers, which is far from universal. CURB-65 cannot be recalled by the vast majority of doctors^27^ and is only recorded for approximately one quarter of patients at initial review.^1^ In summary, while objective assessment of disease severity is an important part of determining the suitable location of care, there are limitations and other important factors that should be considered.

### Which empirical antibiotics to prescribe for patients with CAP?

As with determining the optimal location of care, the choice of empirical antibiotics for patients with CAP is dependent on careful assessment of the cost-benefit of treatment. The potential benefits of appropriate antibiotic choice include reduced morbidity and mortality, shorter duration of symptoms and reduced utilisation of healthcare resources. Whereas inappropriate antibiotic prescribing can increase susceptibility to hospital-acquired infections and can contribute to antimicrobial resistance. The costs and benefits are unique to each individual and each antibiotic or antibiotic combination.

In the UK, choice of empirical antibiotic is recommended on the basis of pneumonia severity, derived from the CURB-65 score and clinical judgement.^18 21^ These recommendations are based on clinical risk; the most unwell patients are prescribed the broadest coverage to reduce the probability of treatment failure. The logic of this stance is reasonable, but strong evidence of clinical effectiveness and safety is limited.^28^ Empirical coverage for atypical organisms is only provided for patients with moderate and severe CAP in the UK, but patients with mild CAP are at the greatest risk of pneumonia secondary to atypical organisms.^28^ In the USA, empirical antibiotics are also recommended on the basis of pneumonia severity, although IDSA/ATS criteria are used rather than CURB-65.^11^ There is also an element of personalisation recommended. Patients with risk factors for *Pseudomonas aeruginosa* or *methicillin-resistant Staphylococcus aureus* (MRSA) that have been locally validated are recommended to receive extended coverage. Similarly, European guidelines suggest using risk factors based on local epidemiology and previous colonisation to guide empirical prescribing for patients with severe CAP.^12^

The success of empirical antibiotic therapy is partly dependent on adequate coverage of the potential causative organisms. CAP is caused by a spectrum of bacteria, viruses and fungi, the distribution of which varies geographically and temporally. *Streptococcus pneumoniae* is the most frequent cause of CAP worldwide, and in the UK.^29^,^31^ It is usually sensitive to narrow-spectrum antibiotics but this is complicated by antimicrobial resistance; approximately 2% of *S. pneumoniae* bloodstream isolates are resistant to amoxicillin and 5% of isolates are resistant to macrolides in the UK.^32^ Additionally, some organisms that cause CAP are routinely resistant to narrow-spectrum antibiotics including *P. aeruginosa*, *MRSA* and *extended-spectrum beta-lactamases*. Several algorithms have been developed to predict the likelihood of CAP caused by drug-resistant organisms.^33^,^39^ However, there is heterogeneity in how these algorithms define drug-resistant organisms, and few have been validated in clinical practice.

While maximising the likelihood of adequate antibiotic coverage is an important aspect of optimal prescribing, there are invariably several antibiotics that could provide adequate coverage. The ideal antibiotic should have a focused narrow-spectrum, limited side effects and robust clinical evidence to support its efficacy. Unfortunately, evidence to support antibiotic prescribing in CAP is limited. All recommendations related to choice of empirical antibiotics for patients in hospital with CAP in the BTS guidelines are based on a formal combination of expert views, at best.^18^ More recently published NICE guidelines in the UK and ATS/IDSA guidelines in the USA do make recommendations based on high-quality evidence, but there are significant limitations and the recommendations of these guidelines differ despite similar publication time.^11 21^ There is randomised controlled trial (RCT) evidence for several antibiotic combinations, but these are not comprehensive and only offer insights into antibiotic effectiveness in a particular setting at a single point in time. This evidence also needs to be considered in context with other factors such as the longer-term adverse effects of antibiotics and their contribution to antimicrobial resistance. For example, fluoroquinolones are subject to a Medicines and Healthcare products Regulatory Agency Drug Safety Update recommending their prescription only when other commonly recommended antibiotics are inappropriate.^40^ Finally, while there is a precedent for personalised empirical antibiotic prescribing in CAP,^41^ such approaches have not been evaluated in RCTs.

## Clinical decision support in practice

There are several areas in the evaluation and treatment of CAP that may be amenable to improvement through the implementation of CDSS. These include improving adherence to antibiotic prescribing guidelines, and using individual patient data to personalise decisions relating to the appropriateness of hospital admission or discharge, and the use of antibiotics and adjunctive therapies.

### Supporting care setting selection in CAP with clinical decision support

As outlined above, severity assessment scores recommended to guide initial management decisions are poorly recalled and underused. Fundamentally, CDSS are a vehicle to communicate information, obviating the need for clinicians to memorise severity scores, irrespective of their complexity. There is evidence that CDSS have been used successfully to this end; following the implementation of a CDSS in the USA that included an automated severity assessment using an electronic adaption of CURB-65, outpatient treatment of patients with CAP from the emergency department increased significantly.^42^ This CDSS also presented the IDSA/ATS minor severe CAP score to providers, and patients with a score of greater than 3 were recommended to receive treatment on ICU. Following implementation, there was a reduction in utilisation of ICU without evidence of harm.^43^ There is less evidence supporting the use of CDSS to guide decision making surrounding discharge of patients admitted to hospital. Such decisions depend on disease severity and trajectory, the likelihood of complications and the availability of support at home if required. While CDSS has the potential to quantify and triangulate this information to support decision making, current evidence for its effectiveness in this context is limited.

### Supporting prescribing decisions in CAP with clinical decision support

As previously mentioned, adherence to antibiotic prescribing guidelines in CAP is frequently suboptimal. The implementation of CDSS has been associated with substantial improvements in adherence to guidelines for the initial choice of empiric antibiotics in CAP.^42 44^ CDSS have also been developed and implemented to guide antibiotic choice based on individual risk of drug-resistant organisms, in keeping with recommendations in European and US guidelines.^39 45^ One such study used a prompt within the computerised provider order entry system to recommend standard spectrum antibiotics, rather than extended spectrum antibiotics, for patients with a low risk of multidrug-resistant organisms.^39^ This prompt was evaluated as part of a large cluster-randomised trial including 59 hospitals and 96 451 patients in the USA and reduced extended spectrum prescribing by over 25% while maintaining clinical safety. A separate study integrated the Drug-Resistance in Pneumonia score in an electronic CDSS, replacing the existing CDSS that used healthcare-associated pneumonia logic. Implementation of the updated CDSS was associated with a relative reduction in broad-spectrum antibiotic prescribing of 28%. Together, these provide evidence that CDSS can be used as a powerful tool to encourage personalised antimicrobial stewardship in CAP.

In addition to antibiotic prescribing, CDSS may be used to support prescribing decisions of immunomodulatory therapies in CAP. There is evidence to support the use of hydrocortisone in patients with severe CAP in the ICU,^46^ and in UK guidelines, it is now recommended that corticosteroids are considered for patients with high-severity CAP.^21^ It has also been suggested that certain subgroups of patients may respond differently to steroid treatment.^47^ As this evidence base evolves, CDSS could be used to facilitate decision making surrounding the prescription of immunomodulatory agents in CAP.

### The future of clinical decision support in CAP

**Figure 2:**
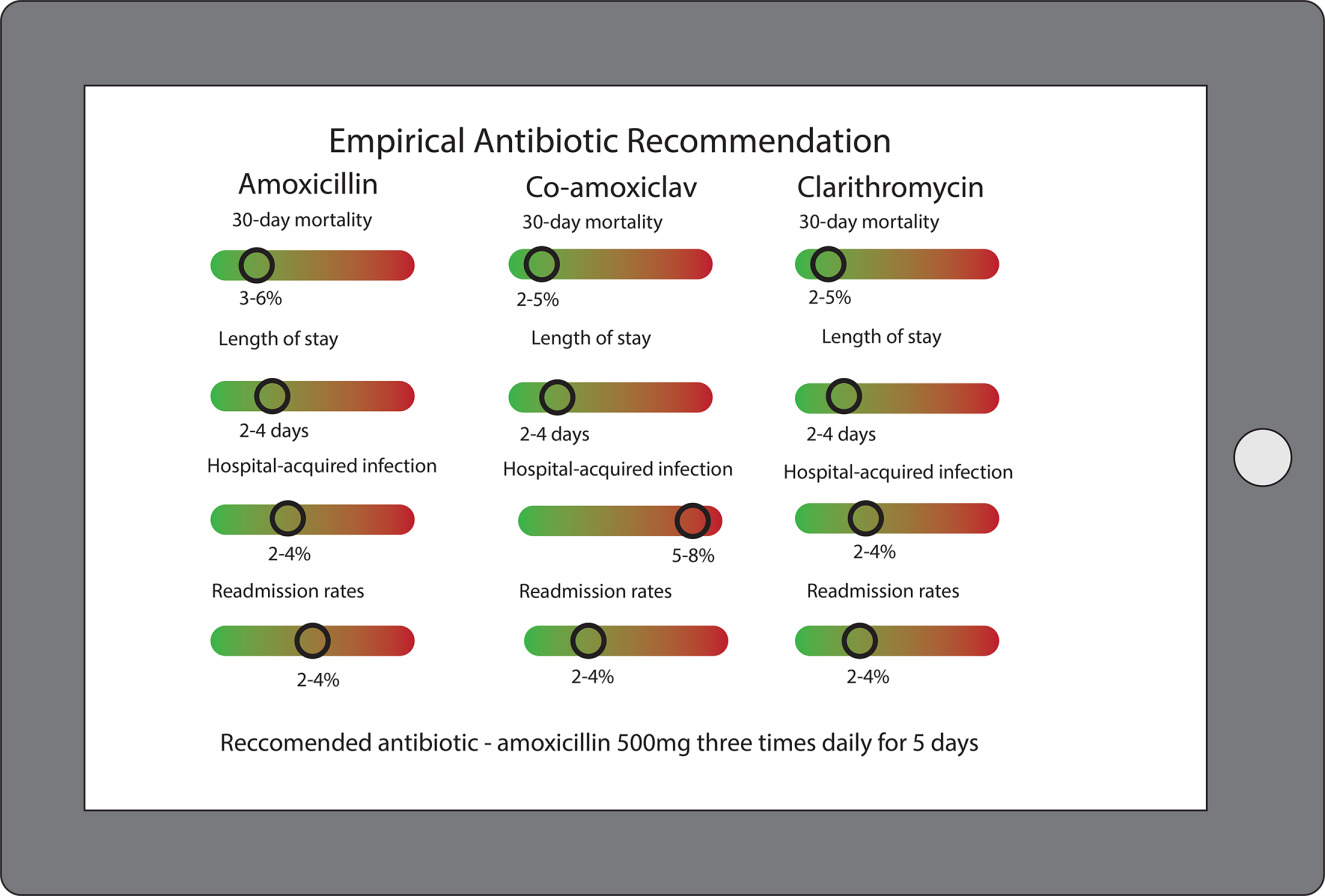
Clinical Decision Support Software (CDSS) example. This figure shows how personalised risk prediction could be incorporated within CDSS to communicate information to patients and optimise antibiotic decision-making.

The CDSS described above varies in their scope and complexity. Some guide decision making across several domains of CAP investigation and management, whereas others have a single well-defined function. It is possible that the greatest benefit of CDSS in CAP may arise from those that are able to triangulate multidimensional data to support personalised decision making, rather than comparatively simple rules-based CDSS. Future CDSS in CAP should reflect the clinical decision making process. Most clinical decisions are based on the judgement that the chosen treatment or decision offers the most favourable risk-benefit profile. For example, the optimal empirical antibiotic or antibiotic combination for an individual depends on their likely outcome, given a range of possible antibiotics, across several outcomes. Causal machine learning could be used to provide outcome predictions under different treatment options using individual patient data, and these could be communicated through a CDSS to guide decision making.^48^ A hypothetical CDSS optimised for empirical antibiotic prescribing in CAP is provided below in figure 2. However, it is important that accurate prediction is not conflated with clinical effectiveness and safety. All predictive models intended to influence treatment decisions should be evaluated in clinical practice with data collected pertaining to all important outcomes.

## Discussion

While considerable work is required to establish a firmer evidence base to support the initial management of CAP, CDSS have significant potential. The CDSS discussed above provides high quality evidence that CDSS can positively influence antimicrobial stewardship in CAP.^39^ At present, this is the only outcome supported by high quality evidence at low risk of bias. There are other potential benefits of CDSS for CAP management; implementation has been associated with improvements in process outcomes such as guideline adherent antibiotic prescribing, setting of care and clinical outcomes including mortality.^49^ However, improvements in these outcomes require further validation in studies with robust methodology.

There are several unintended consequences of CDSS implementation that are widely reported but inconsistently measured when CDSS are evaluated. It is reported that up to 95% of CDSS alerts are inconsequential,^50^ which can cause alert fatigue, making clinicians less likely to use information from clinically important alerts. While the pre-existing CDSS infrastructure will inevitably influence the impact of newly implemented CDSS, it is rarely reported. CDSS may also cause delays in medication administration, and these have been reported to have substantial negative impacts in some cases.^51^ It is vital that CDSS are co-designed with end users, evaluated rigorously offline prior to implementation and are subject to ongoing quality monitoring with de-implementation or adaptation where necessary.

It is notable that most studies evaluating the impact of CDSS implementation in pneumonia are conducted in the USA, where the organisational context and antibiotic prescribing culture differ from other countries such as the UK. In the USA, multiple hospitals are often owned by a single private healthcare company and use the same electronic health record (EHR), making implementation and evaluation of CDSS comparatively simple compared with other countries such as the UK. In the UK, National Health Service (NHS) Trusts often use different EHR providers and operating systems, making wide-scale implementation and evaluation of CDSS challenging. A Digital Maturity Assessment in the UK in 2023 found that while 90% of NHS Trusts used an electronic patient record, only 10–30% had functions such as integrated prescribing and record sharing with other hospitals.^52^ A systematic review exploring the impact of CDSS on antibiotic prescribing did not identify any that had been implemented in secondary care in the UK or Europe that were specific to CAP.^49^ A small number of CDSS have been implemented in Europe for antibiotic prescribing more broadly, but these studies have too few patients to make inferences about their utility in CAP. Given that the current evidence base is predominantly based on studies conducted in the USA, results may not be directly transferable. Further prospective evaluation in the UK will be required as digital maturity increases and the use of CDSS increases.

Interest in the personalised management of CAP using individual patient data is likely to grow as routinely collected healthcare data becomes more readily available and digital maturity advances. It is vitally important that these algorithms are evaluated prospectively in clinical practice using a wide range of clinical and health economic outcomes to firmly establish their benefit, safety and cost effectiveness. It is also essential that guideline committees do not take an algorithmic-centric view of their use. Often, the clinical impact of a CDSS is attributed to the underlying algorithm or clinical score alone. In reality, the benefits and risks of the CDSS are inseparable from their integration within the EHR and clinical workflow, and how outputs are communicated with clinical decision makers. Where evidence for guidelines is derived from studies using CDSS, guideline committees should consider making recommendations about local implementation and integration of CDSS into the clinical workflow, rather than only recommending the use of the underlying algorithm.

Increasing complexity of CDSS also brings with it several technical, logistical and ethical challenges that developers, implementors and users need to be cognisant of. NHS Trusts across the UK are at differing stages of digital maturity and investment is needed to develop capacity and infrastructure to ensure that all regions can benefit from CDSS. The negative consequences of health data poverty are also well recognised^53^ and the use of datasets that are representative of the target population is essential to ensure health inequalities are not worsened.

## Conclusion

In conclusion, CAP is a significant burden on the health of populations across the world. Despite this, there has been relative underinvestment in CAP research and the evidence base to guide decision making for the initial management is incomplete. CDSS are a powerful tool to communicate complex information and promote behaviour change and have significant potential to optimise the decision making of clinicians treating patients with CAP. Future research should aim to establish their clinical effectiveness and safety across a wider range of outcomes relevant to the management of CAP.
